# Mammalian models of extended healthy lifespan

**DOI:** 10.1098/rstb.2010.0243

**Published:** 2011-01-12

**Authors:** Colin Selman, Dominic J. Withers

**Affiliations:** 1 Institute of Biological and Environmental Sciences, University of Aberdeen, Aberdeen AB24 2TZ, UK; 2 Metabolic Signalling Group, Medical Research Council Clinical Sciences Centre, Imperial College, London W12 0NN, UK

**Keywords:** insulin signalling, ageing, nutrient sensing, target of rapamycin

## Abstract

Over the last two centuries, there has been a significant increase in average lifespan expectancy in the developed world. One unambiguous clinical implication of getting older is the risk of experiencing age-related diseases including various cancers, dementia, type-2 diabetes, cataracts and osteoporosis. Historically, the ageing process and its consequences were thought to be intractable. However, over the last two decades or so, a wealth of empirical data has been generated which demonstrates that longevity in model organisms can be extended through the manipulation of individual genes. In particular, many pathological conditions associated with the ageing process in model organisms, and importantly conserved from nematodes to humans, are attenuated in long-lived genetic mutants. For example, several long-lived genetic mouse models show attenuation in age-related cognitive decline, adiposity, cancer and glucose intolerance. Therefore, these long-lived mice enjoy a longer period without suffering the various sequelae of ageing. The greatest challenge in the biology of ageing is to now identify the mechanisms underlying increased healthy lifespan in these model organisms. Given that the elderly are making up an increasingly greater proportion of society, this focused approach in model organisms should help identify tractable interventions that can ultimately be translated to humans.

## 1. Introduction

The last 200 years has seen an astonishing increase in human life expectancy in the developed world, with an estimated 30 years added to average life expectancy since the turn of the twentieth century [1]. This increase has been achieved through factors including better diet, cleaner water and significantly improved preventative medicine and palliative care [2]. The elderly are therefore making up a significantly greater proportion of the population, particularly when allied to low fertility and immigration [1]. Recent projections suggest that approximately 1.6 per cent of the UK population, compared with the current approximately 0.7 per cent, will be over 90 years of age by 2020 [3]. This means that if current trends continue, then the majority of babies born in the UK since 2000 will celebrate their 100th birthday [1]. These rapidly altering demographic profiles will have enormous social, economic and ethical implications because ageing is inexorably linked to physiological decline, loss of independence and decreased quality of life [4]. In particular, the prevalence of cardiovascular disease, dementia, cancer, sarcopaenia, osteoporosis, osteoarthritis and type-2 diabetes all increase significantly with advancing age [4–6].

Obvious practical, ethical and economic obstacles exist to longitudinal studies investigating the determinants of human lifespan. Therefore, much effort in the biology of ageing has employed model organisms like yeast, the nematode worm *Caenorhabditis elegans*, the fruitfly *Drosophila melanogaster* and the mouse [2,7–9]. While it has been established for over 70 years that dietary restriction (DR) extends lifespan in many organisms [10–12], it has only been more recently that ageing and lifespan have been shown to be modulated by genetic factors [2,13–15]. Furthermore, many age-related pathophysiological processes relevant to the diseases of ageing in humans can be modelled in such organisms.

Specific mutations in the insulin/insulin-like growth factor (IGF) signalling (IIS) pathway extend lifespan in model organisms [7–9,13,16–19]. Polymorphisms in several IIS and growth hormone (GH)-related genes correlate with human longevity [20–22], and attenuated IIS may underlie the long life of GH/GH receptor-deficient dwarf mice (e.g. Ames (*Prop1*^*df/df*^), Snell (*Pit1*^*dw/dw*^), Little (*Ghrhr*^*lit/lit*^), growth hormone receptor knockout (*GHR-KO*) [23]). The target of rapamycin (TOR) pathway also plays a key and conserved role in longevity control [24–29]. It is clear that understanding how exactly the IIS, GH and mTOR signalling pathways interact with one another to increase lifespan and healthspan is a key challenge to future research.

The primary objective of biology of ageing research is to identify what underpins ageing in order to generate interventions that attenuate age-related pathology and consequently improve healthy lifespan in humans. Therefore, model organism studies that show both extended lifespan and improved health over this long life are crucial if we hope to achieve this. This review will therefore examine those studies of genetically modified mice demonstrating evidence of extended healthy lifespan through the positive effects on age-sensitive biomarkers and/or resistance to age-related pathology (table 1). Our approach is to describe in turn the various pathophysiological processes and diseases relevant to ageing in humans that are ameliorated in long-lived genetic mice. However, it should be noted that in many cases a limited number of pathologies have been examined, with relatively few long-lived mutants having undergone detailed examination in a broad range of disease parameters.

**Table 1.** Attenuated ageing-related decline in various phenotypic parameters in genetic mouse models of healthy ageing. (Long-lived genetic model and primary reference. 1, Homozygous ribosomal protein S6 kinase 1 knockout [29]; 2, homozygous insulin receptor substrate 1 knockout [19]; 3, homozygous fat-specific insulin receptor knockout [16]; 4, heterozygous insulin receptor substrate 2 knockout ([44], but see also [45]); 5, heterozygous insulin-like growth factor 1 receptor knockout [46]; 6, mutation of *Prop1*^*df/df*^ [122]; 7, mutation of *Pit1*^*dw/dw*^ [41]; 8, homozygous growth hormone receptor (Laron) knockout [123]; 9, homozygous regulatory subunit of protein kinase A knockout [48]; 10, combined telomerase reverse transcriptase and p53, p16 and p19ARF transgenic [50]; 11, homozygous pregnancy-associated plasma protein A knockout [93]; 12, homozygous macrophage migration inhibitory factor knockout [94]; 13, homozygous type 5 adenylyl cyclase knockout [68]; 14, mitochondrial targeted catalase transgenic [77]; 15, heterozygous glutathione peroxidase 4 knockout [74]; 16, metallothionein-IIa transgenic [79]; 17, homozygous mammalian proto-oncogene 66K knockout [80].)

ref.	mouse model	phenotypic parameter						
		metabolism	cognition	bone	eye	heart	cancer	immunity
1	*S6K1*^*−/−*^	+	+	+				+
2	*Irs1*^*−/−*^	+	+	+				+
3	FIRKO	+						
4	*Irs2*^*+/−*^	+						
5	*Igf1r*^*+/−*^	+						
6	Ames	+	+				+	
7	Snell			+	+		+	+
8	*GHR-KO*	+	+		+		+	+
9	RIIβ	+				+	+	
10	*Sp53/Sp16/SArf/TgTert*	+	+					+
11	PAPP-A-KO						+	+
12	MIF-KO						+	
13	*AC5 KO*			+		+		
14	MCAT				+	+	+	
15	*Gpx4*^*+/−*^						+	
16	MT					+		
17	*p66*^*sch−/−*^					+		

## 2. Mutations in individual genes that extend healthy lifespan in mammals

### (a) Maintenance of a youthful metabolic profile in long-lived mice

Ageing in humans is associated with greater adiposity, particularly visceral fat, higher body mass index and a significant loss in lean mass [30–32]. Commonly associated with these age-related changes in body composition are impairments in glucose tolerance and insulin sensitivity. Elevated adiposity and insulin resistance are significant risk factors for type-2 diabetes, coronary heart disease and stroke. In addition, insulin resistance is linked to higher colon, liver and pancreatic cancer incidence [33], more aggressive breast cancer tumours [34] and Alzheimer's disease (AD) development [35].

The most widely studied phenotypic parameters in long-lived mice are those associated with body composition and glucose/insulin homeostasis. It is well established that long-lived GH/GH receptor dwarf mice are highly insulin sensitive [36–38]. Indeed, the absence of lifespan extension in *GHR-KO* mice following DR [36] or intermittent fasting [39] is suggested to be because these dietary interventions cannot further increase insulin sensitivity in these animals [40]. However, long-lived Snell dwarf mice are obese and hyperleptinaemic in old age [41], and have unaltered fasting (3–6 h) insulin and glucose levels relative to control mice at 20–23 months of age [41,42]. Percentage fat mass, normalized to total body mass, was also elevated in *GHR-KO* mice over a 2 year period relative to controls, with the increased adiposity more apparent in males [43].

Long-lived fat-specific insulin receptor knockout (FIRKO) mice are lean, have reduced total-body triglyceride levels and show resistance to age-related deterioration in glucose tolerance compared with wild-type (WT) controls [16]. Insulin receptor substrates 1 and 2 are key intracellular effectors of the IIS receptors [19]. Mice heterozygote for insulin receptor substrate 2 (*Irs2*^*+/−*^) have been described as long-lived in one study [44], but not in another despite using the same mouse model [45]. Nonetheless, both male and female *Irs2*^*+/−*^ mice were significantly more insulin sensitive at approximately 22 months of age relative to controls in the earlier study, despite no differences observed between genotypes at two months of age [44]. Reportedly, long-lived female mice heterozygote for the IGF-1 receptor (*Igf1r*^*+/−*^) ( [46], but see also [47]) are also slightly more glucose tolerant compared with control animals [46]. Recently, we have shown that 600-day-old female mice null for ribosomal protein S6 kinase 1 (*S6K1*^*−/−*^), a downstream effector of mTOR signalling and IIS, are lean, hypoleptinaemic, glucose tolerant and insulin sensitive (assessed by the updated homeostasis model, HOMA2; [29]). These improvements are despite *S6K1*^*−/−*^ mice being glucose intolerant at eight weeks of age [29].

Mice null for RIIβ, a regulatory subunit of protein kinase A, are long-lived, and resistant to both age-related obesity and hyperleptinaemia [48]. These mice also have lower fasting blood glucose levels at 24 months of age compared with WT littermates [48]. Male RIIβ mutants were more insulin sensitive at young (two to five months) and old (18 months) ages relative to controls, although young females were not [48]. Studies examining the role of the cellular reverse transcriptase telomerase (TERT) during ageing have been complicated by the cancer-promoting effects of telomerase [49]. However, over-expression of Tert in the background of enhanced cancer resistance (enhanced expression of p53, p16 and p19ARF) increased lifespan in *Sp53/Sp16/SArf/TgTert* transgenic mice [50]. These animals were also protected against age-related deteriorations in glucose tolerance between 30 and 76 weeks of age [50].

However, several other studies demonstrate that insulin sensitivity *per se* is not a prerequisite for a long and healthy lifespan. For example, long-lived female insulin receptor substrate 1 null (*Irs1*^*−/−*^) mice are both insulin and IGF-1 resistant at 450 days of age [19]. However, remarkably, these animals are protected against an age-related deterioration in glucose tolerance seen in WT animals because of lifelong beta-cell compensation and associated hyperinsulinaemia [19]. It should also be noted that despite this insulin-resistant phenotype, *Irs1*^*−/−*^ mice are lean and hypoleptinaemic at 450 days and 700 days of age [19]. Long-lived heterozygote brain-specific IGF-1 receptor knockout mice (bIGF1RKO^+/−^) had greater adiposity, hyperleptinaemia and impaired glucose tolerance at 10 months of age compared with WT mice [18]. Insulin resistance and glucose intolerance were also observed in long-lived brain-specific IRS2 heterozygote and homozygote (*bIrs2*^*+/−*^, *bIrs2*^*−/−*^) mice [44]. Finally, mice with over-expression of *Klotho*, a single-pass transmembrane protein, are long-lived but insulin and IGF-1 resistant [51].

### (b) Preserved cognitive and motor functions in long-lived mice

Age itself is the greatest risk factor for cognitive decline and dementia [5]. Brain-related pathologies will have increasingly important implications to healthcare, governmental policy and society, given that recent estimates predict that AD cases in the USA alone will rise from 377 000 cases in 1995 to around one million by 2050 [52].

Undoubtedly, the best studied long-lived mice with regard to cognitive function and ageing are the GH/GH receptor dwarfs, with several studies indicating preserved cognitive function in these mice at old age [53–55]. For example, old (17–20 months) *GHR-KO* and Ames mice (19–21 months) had improved learning and memory compared with age-matched WT controls [54,55]. Exactly why this is the case is currently unknown, but intriguingly Ames mice have been shown to have increased hippocampal neurogenesis in adulthood [56,57].

Several other studies suggest that delayed age-related cognitive decline exists in long-lived mice. For example, female *Irs1*^*−/−*^ mice at 450 days of age showed better motor/neurological function, as assessed by forced motor activity performance on a rotating rod (rotarod) apparatus, compared with WT mice [19]. Importantly, no improvement was seen in young (80-day-old) *Irs1*^*−/−*^ mice, suggesting that the better rotarod performance in old age was not due to the dwarf phenotype. An enhancement in rotarod performance was also demonstrated in female *S6K1*^*−/−*^ mice at 600 days of age [29]. In addition, these mice had enhanced overall general activity and exploratory drive performance during open-field testing at this age [29]. *Sp53/Sp16/SArf/TgTert* mice at approximately 12 months of age also demonstrated better neuromuscular coordination during a tightrope test [50], and *bIrs2*^*+/−*^ and *bIrs2*^*−/−*^ mice were significantly more active than controls at 22 months of age [44].

Long-lived mice also appear more resistant to the sequelae of neurodegenerative conditions such as AD. For example, hippocampal slices from adult Ames mice are significantly more resistant to β-amyloid (Aβ) toxicity compared with controls [58]. AD is associated with altered neuronal insulin signalling [59], and several studies have investigated the effect of altered insulin signalling on AD progression. Mice expressing the Swedish mutation of amyloid precursor protein (APP^SW^, Tg2576) and crossed with long-lived bIGF1RKO^+/−^ mice [18] were rescued from APP^SW^-induced early mortality and had significantly reduced Aβ accumulation at 60 weeks of age [59]. In addition, global IRS-2 deficiency in this AD model completely reversed early mortality and delayed Aβ accumulation [59]. In agreement, global IRS2 deficiency in APP^SW^ mice ameliorated Aβ pathology and improved several behavioural parameters despite increasing tau phosphorylation [60]. Interestingly, this is despite *Irs2*^*−/−*^ mice of both sexes being significantly short-lived [19]. Mice expressing two AD-linked transgenes and crossed with *Igf1r*^*+/−*^ mice [46] were protected against several AD-related pathologies and impaired cognitive function [61]. Rapamycin, an inhibitor of the mTOR pathway, treatment significantly increases lifespan in mice [25] and was recently shown to attenuate cognitive deficits, Aβ pathology and tau pathology in two different AD mouse models [62,63].

### (c) Delayed bone loss in long-lived mice

Osteoporosis, which clinically presents as reduced bone mass and altered bone structure, is a significant public health issue. For example, osteoporosis-related fractures, particularly hip fractures, are a major cause of morbidity and mortality in humans [64]. Clinical studies also suggest that there is overlap between several common disease mechanisms underlying osteoporosis and cardiovascular disease, including chronic inflammation, insulin resistance and obesity [65].

Mice also are prone to significant losses in bone volume with advancing age (e.g. [66]). Several studies examining long-lived mice have used a cross-sectional approach to show simultaneous preservation of bone function and quality with age. We have demonstrated, using micro-computed tomography, that female *Irs1*^*−/−*^ mice at 450 and 700 days of age are resistant to age-related bone dysfunction [19]. *Irs1*^*−/−*^ mice had greater cancellous bone volume, increased trabecular number and reduced trabecular separation compared with age-matched controls. Interestingly, this preservation in bone quality during ageing was seen despite young *Irs1*^*−/−*^ mice being osteopenic [67]. Furthermore, using the same methodology, we recently showed that 600-day-old female *S6K1*^*−/−*^ mice also had greater tibial bone volume and trabecular number compared with age-matched WT controls [29]. A preservation in bone volume with age was also reported in mice lacking type 5 adenylyl cyclase (*AC5 KO*), a key catalytic enzyme in the synthesis of cyclic adenosine monophosphate from adenosine triphosphate [68]. Female *AC5 KO* mice at 23 months of age had greater femoral bone density and calcification, less evidence of healing stress fractures and improved bone strength compared with controls [68]. Interestingly, tail tendons from young (four to eight months) and old (16–19 months) Snell mice are more resistant to urea-induced collagen denaturing, and Snell mice are protected against articular ageing and osteoarthritis [41,69]. Preservation of skeletal function during ageing is therefore often seen in long-lived mouse models.

### (d) Attenuated visual deterioration in long-lived mice

Visual impairment and visual loss caused by several pathologies including macular degeneration, glaucoma and cataracts increase significantly in humans with advancing age [70]. These visual impairments may also exacerbate additional age-related pathologies including metabolic dysfunction, cardiovascular disease, insomnia, depression and impaired cognition through disruption in circadian photoreception [71].

Mice show comparable age-related increases in cataracts to humans, with similar regions of the lens affected. For example, C57BL/6 mice display a highly significant increase in the degree of cataract severity between six and 28 months of age [72]. Using a slit lamp protocol, it has been shown that both *GHR-KO* mice [72] and Snell dwarf mice [73] are resistant to age-related cataracts, with no differences in cataract levels relative to WT controls reported in *GHR-KO* mice at six months of age [72]. However, delayed cataract formation does not appear to be universal among long-lived mutant mice, as no difference was reported between long-lived glutathione peroxidase 4 heterozygous knockout mice (*Gpx4*^*+/−*^) and controls at 25 months of age [74].

### (e) Improved cardiac function in long-lived mice

Cardiovascular disease is a primary cause of mortality in humans worldwide, with its risk increasing significantly with advancing age [75]. Cardiovascular disease *per se* is not thought to be a prominent cause of mortality in most laboratory mouse strains. However, many of the age-related deteriorations in cardiovascular function observed in humans are also observed in mice [76]. Consequently, there is good evidence that aspects of cardiovascular ageing are significantly attenuated in long-lived mice.

It has been demonstrated that *AC5 KO* mice, for example, are resistant to age-related myocardial fibrosis and left ventricular (LV) hypertrophy [68]. These mice also have reduced cardiac apoptosis and smaller myocyte cross-sectional area at 20–30 months of age compared with WT mice. Male RIIβ null mice have preserved cardiac function and less evidence of LV hypertrophy at 24 months of age [48]. Long-lived mice over-expressing human catalase within their mitochondria (MCAT mice) [77] were also highly resistant to a range of cardiac-associated pathologies, including arteriosclerosis, cardiomyopathy, impaired diastolic function and LV hypertrophy at 20–25 months of age [76,77]. Interestingly, age-dependent cardiomyopathy in mice carrying a homozygous mutation in the exonuclease-encoding domain of mitochondrial polymerase gamma (Polg^m/m^ mice) was ameliorated when Polg^m/m^ mice were crossed with MCAT mice [78]. Transgenic mice over-expressing human metallothionein-IIa, a heavy metal-binding antioxidant, in cardiac tissue are long-lived and protected against both age-related diastolic dysfunction and decreased cardiac contractile reserve capacity [79]. Long-lived mice null for the cytoplasmic adaptor protein p66^shc^ (*p66*^*shc−/−*^) are long-lived [80] and resistant to age-dependent reactive oxygen species-mediated endothelial dysfunction [81]. *p66*^*shc−/−*^ mice were protected against angiotensin II-induced LV hypertrophy, associated cardiomyocyte and endothelial cell apoptosis [82]. Long-lived mice null for either pregnancy-associated plasma protein A (PAPP-A-KO) [83], a metalloproteinase that degrades IGF-binding proteins, or p66^shc^ [84] are protected against high fat diet-induced atherosclerosis when maintained in an apolipoprotein E-deficient (ApoE) background.

### (f) Protection against cancer in long-lived mice

Many cancers, including breast, prostate and colorectal, increase with advancing age, with the vast majority of cancer cases seen in people over 60 years of age [85]. The risk of mortality from cancer also increases in an age-dependent manner [85,86], although both incidence and mortality rate apparently plateau and subsequently decline after 90 years of age [86]. Cancer is the primary cause of death in most mouse strains, although tumour type appears highly strain specific [87]. For example, the C57BL/6 strain commonly used in ageing research is particularly prone to lymphosarcoma [74,88]. Significant effort has focused on whether interventions that extend lifespan impact on cancer incidence and progression, with most studies examining end of life pathology.

Several studies have investigated whether long-lived GH/GH receptor-deficient dwarf mice are protected against cancer [89,90]. In Ames dwarfs, age-related neoplastic disease was delayed and adenocarcinoma severity reduced despite the percentage of tumour-bearing mice at death being similar to WT mice [91]. However, tumour burden (the number of different tumours) in Ames mice was similar to controls [91]. In contrast, Snell dwarfs had significantly reduced age-related tumour burdens [89] and a non-significant trend (*p* = 0.06) for reduced lymphoma and mammary adenocarcinoma [73]. *GHR-KO* mice also had decreased tumour incidence, reduced tumour burden, less severe pulmonary adenocarcinomas and an age-related delay in fatal neoplastic diseases relative to controls [92].

A comprehensive examination by two pathologists reported that a similar percentage of *Gpx4*^*+/−*^ and control mice (55–60%) had neoplastic disease, although an age-related delay in fatal lymphoma was seen in *Gpx4*^*+/−*^ mice [74]. Gross pathological investigation of 23- to 28-month-old PAPP-A-KO mice revealed significantly reduced tumour burden and no evidence of multiple tumours unlike control mice [93]. RIIβ^−/−^ null mice had less lymphosarcoma and splenic tumours compared with controls, although no differences were observed in hepatic tumours [48]. MCAT mice had unaltered haematopoietic tumour incidence compared with WT controls, but a significant reduction in the severity and tumour burden of non-haematopoietic tumours was seen post-mortem [88]. Mice null for the pro-inflammatory cytokine macrophage migration inhibitory factor (MIF-KO) showed less evidence of hemangiosarcomas compared with WT controls [94]. However, we observed no difference in macroscopic tumour incidence at death in female *S6K1*^*−/−*^ compared with controls [29], in agreement with findings in bIGF1RKO^+/−^ mice [18]. Lifespan extension following rapamycin treatment in mice also did not alter the distribution in the presumptive causes of mortality in mice, including tumour incidence [25].

### (g) Youthful immune profile and reduced inflammation in long-lived mice

The presence of a chronic inflammatory state in humans, e.g. elevated pro-inflammatory cytokines and local infiltration of assorted inflammatory cells, underlies several ageing-related diseases, including cancer [6,86]. In mice, alterations in T-cell populations (i.e. greater proportion of naive T cells relative to memory T cells) are predictive of both a resistance to various cancers [95] and longevity [96,97]. However, it should be noted that this assay may not specifically indicate improved immune function (e.g. resistance to infection). In addition, mouse ageing studies by their very nature tend to use specific pathogen-free environments to protect mice from infection. Therefore, under these experimental conditions, definitive measures of improved immune function are consequently difficult to assay. However, functional assays such as viral challenge may be useful in determining whether immune function is maintained in long-lived mice during ageing. Interestingly, intranasal inoculation with influenza (H1N1, PR8) actually increased mortality, increased weight loss and diminished innate immunity in male DR mice [98].

We previously reported that female *Irs1*^*−/−*^ mice at 450 and 700 days of age [19] and female *S6K1*^*−/−*^ mice at 600 days of age [29] had significantly fewer memory and more naive T cells relative to WT mice. This is suggestive of a more youthful immune profile in these animals. Interestingly, the age-related reduction in haematopoietic stem cell number linked to anaemia, impaired response to vaccination and tumourigenesis was reversed in old mice following rapamycin treatment [99].

Aged mice, like humans, are susceptible to several inflammatory pathologies, including dermatitis, gastritis, peritonitis and enteritis [50]. Both male and female *Irs1*^*−/−*^ mice were completely resistant to the age-related ulcerative dermatitis observed in old C57BL/6 mice, whereas 25 per cent of WT mice were afflicted [19]. From 50 weeks of age onwards, long-lived *Sp53/Sp16/SArf/TgTert* mice were protected against age-related thinning of their epidermis and subcutaneous fat layer [50]. These mice also had higher skin keratinocyte telomerase activity, longer hair bulge and interfollicular epidermal telomeres, and higher clonogenic potential of epidermal stem cells at both young and old ages [50]. *Sp53/Sp16/SArf/TgTert* mice also had preserved intestinal tract epithelia during ageing and enhanced resistance to dextran sodium sulphate-induced intestinal ulcers [50]. PAPP-A-KO mice at 18 months of age are resistant to age-dependent thymic atrophy, have a more youthful T-cell profile and bone marrow enriched with thymus-seeding progenitor cells compared with controls [100]. MCAT mice also showed a trend towards less severe systemic inflammation [88]. Aged (27–29 months) Snell dwarfs are resistant to age-related changes in T-cell subsets, with preservation of their immune cell function and fewer P-glycoprotein (anergic)-expressing splenic cells [41]. However, as mentioned previously, the relationship between immune function and ageing appears incredibly complex. For example, long-lived Ames dwarf mice actually show various manifestations of immunodeficiency, in particular those relating to thymic function [101].

## 3. Conclusions

The application of mouse models to help inform what mechanisms underlie healthy ageing in mammals has provided exciting insights into this process but remains experimentally challenging. However, it is evident that despite these paradigm-shifting experiments, we still have no definitive mechanistic explanation of the ageing process, although enhanced resistance to oxidative stress ([102,103] but see also [104,105]), increased xenobiotic metabolism [106,107], altered mitochondrial function [108,109] and enhanced autophagy [110,111], for example, may be key, and not mutually exclusive, candidate mechanisms. Ames dwarf mice, for example, have enhanced paraquat resistance and lower liver and lung F(2) isoprostane levels in old age (14–20 months) compared with controls [112]. Fibroblasts from GH-deficient mice are also highly stress resistance [113,114], with Snell dwarfs fibroblasts showing enhanced antioxidant and base excision repair capacity following combined serum deprivation and paraquat exposure [115]. Therefore, we suggest that it is critical to determine whether parameters including stress resistance and base excision repair capacity are also altered in an age-dependent manner in other long-lived mouse models. Certainly, other mechanisms are also likely to be at work and full consideration is beyond the scope of this review, but are well covered elsewhere (e.g [116–119]).

It is highly evident that using mammalian models to understand what underlies extended healthy lifespan is a rapidly expanding and competitive field of research. This is clearly indicated by the fact that seven long-lived genetic mouse models were described by 2003 [47] but over 20 models have been reported by 2009 [120]. The basic rationale for undertaking these long-term, demanding and expensive studies is to determine whether increased lifespan translates to increased healthspan in later life. Therefore, those studies that simultaneously measure lifespan, assay biomarkers predictive of lifespan [121] and examine age-related pathology are likely to be our best hope of identifying tractable interventions that can ultimately be applied to humans. In particular, we feel that future ageing studies should pay particular attention to late age health.
